# Job satisfaction among family medicine residents: A cross-sectional study

**DOI:** 10.1192/j.eurpsy.2025.1588

**Published:** 2025-08-26

**Authors:** M. Hajji, N. Rmadi, A. Hrairi, F. Dhouib, I. Ben Hnia, M. Hajjaji, K. Jmal Hammami

**Affiliations:** 1 Family medicine department, University of Sfax; 2 Occupational medicine department, Hedi chaker university hospital, University of Sfax, Sfax, Tunisia

## Abstract

**Introduction:**

Job satisfaction reflects on overall life quality involving social relationship, family connection and a feeling of enjoyment or fulfillment specially for family medicine residents’ contentedness with their job.

**Objectives:**

This study aims to assess factors associated to job satisfaction in a population of Tunisian family medicine residents.

**Methods:**

We conducted a cross-sectional study from January to July 2024. Family medicine residents participated in the survey through a Google Forms questionnaire. They were queried about their sociodemographic information and work conditions, including daily working hours, the number of night shifts per month, and the average number of patients seen each day. To assess participants’ job satisfaction, we used the validated physician’s job satisfaction scale which covered five domains: patient care, burden, income-prestige, personal reward, and professional relations.

**Results:**

We enrolled 108 family medicine residents (65 women and 43 men) with an average age of 26 ± 2 years [22-37 years]. The average daily working hours were 6.62 hours, with an average of 15 patients seen each day. Additionally, the average number of night shifts completed per month was 5. Positive correlations were found between age and satisfaction with time spent with family friends or leisure activities (p=0.001, r=0.3), burden’s average mean (p=0.01, r=0.2) and opportunities for continuing medical education (p=0.03, r=0.1). However, a negative correlation was found between satisfaction with professional relations and the number of night shifts per month (p=0.01, r= - 0.2).

**Conclusions:**

This research highlights the critical factors influencing job satisfaction among family medicine residents in Tunisia, with a particular focus on their working conditions. Addressing these issues is essential for enhancing the overall satisfaction of these residents. By creating a supportive work environment, we can ultimately improve patient care and treatment outcomes.

**Disclosure of Interest:**

None Declared

